# Catheter Displacement into Inferior Epigastric Vein Causing Local Phlebitis and Cellulitis

**DOI:** 10.1155/2012/492594

**Published:** 2012-07-02

**Authors:** Noriko Hattori, Hidenori Hattori, Kazushi Takahashi, Norihiro Suzuki, Kazuo Kishi

**Affiliations:** ^1^Department of Plastic and Reconstructive Surgery, Keio University Hospital, 35 Shinanomachi, Shinjuku-ku, Tokyo 160-8582, Japan; ^2^Department of Neurology, Saitama Manicipal Hospital, Saitama 336-8522, Japan; ^3^Department of Neurology, Keio University Hospital, 35 Shinanomachi, Shinjuku-ku, Tokyo 160-8582, Japan

## Abstract

Catheter insertion for intravenous hyperalimentation is a commonly and widely used clinical technique. When compared with the incidence of complications associated with insertions into the internal jugular vein or the subclavian vein, complications associated with insertions into the femoral vein are less frequent. In this paper, we describe a very rare complication of femoral vein catheter insertion—namely, catheter displacement into the inferior epigastric vein.

## 1. Introduction

The technique of catheter insertion for intravenous hyperalimentation has been commonly and widely used since it was first reported in 1967 [1]. However, there have also been reports of complications associated with this procedure. These include infection, air embolism, thrombosis, and damage to the catheter itself. Catheter migrations into smaller veins were often reported in neonates, but have been rarely noted in adults [2]. 

We report the case of a 77-year-old man with a cerebral haemorrhage who had local phlebitis and cellulitis caused by catheter displacement into the inferior epigastric vein.

## 2. Case Report

**Figure 1:**
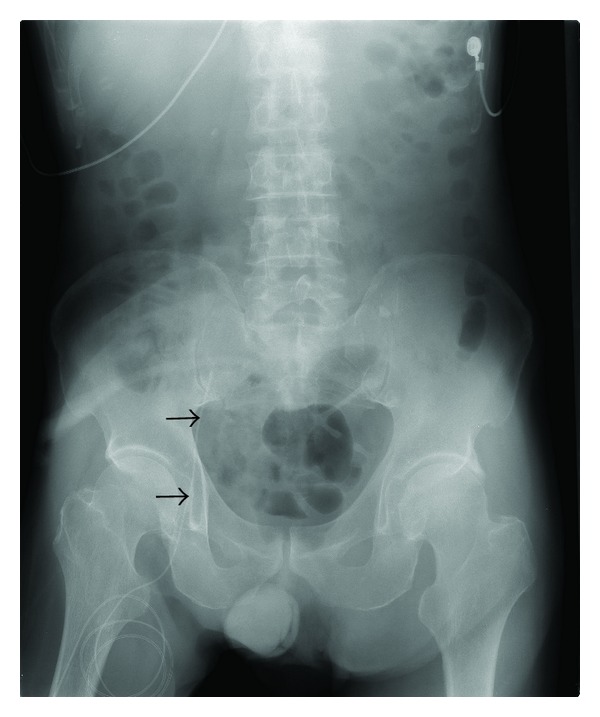
A plain radiograph confirms the position of the catheter.

**Figure 2:**
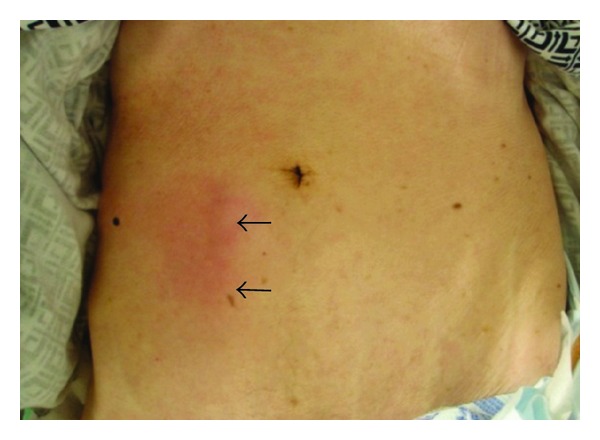
A photograph of the abdomen demonstrates subcutaneous swelling in the right lower quadrant of the abdomen (see area adjacent to black arrows).

**Figure 3:**
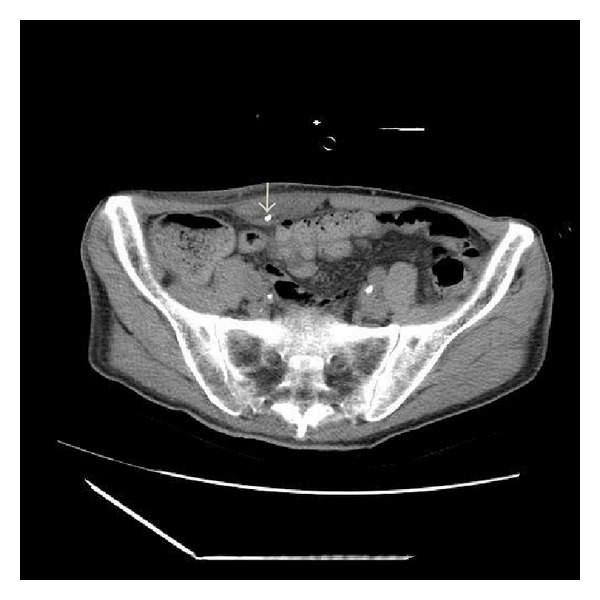
Axial computed tomography reveals the catheter in the right inferior epigastric vein as well as swelling of the right lower rectus abdominis muscle and adjacent subcutaneous tissue.

A catheter was inserted for intravenous hyperalimentation into the right femoral vein of a 77-year-old man with a cerebral hemorrhage. Hyperalimentation was started after the position of the catheter was confirmed by a plain radiograph (Figure 1). Antibiotics (PAPM/BP) had been used for the laryngitis by the otolaryngologist for 9 days and stopped. The patient developed fever, subcutaneous swelling, and redness in right lower quadrant of the abdomen 11 days later (Figure 2). An axial abdominal computed tomographic (CT) scan for suspected phlebitis and cellulitis revealed that the catheter was located in the right inferior epigastric vein. Swelling of the adjacent right lower rectus abdominal muscle and subcutaneous tissue was also evident (Figure 3). Intravenous hyperalimentation was stopped and the catheter was removed. Subsequently, the subcutaneous swelling and clinical redness disappeared within a few days. We did not use any antibiotics for the cellulitis.

## 3. Discussion

Catheter displacements into small veins have often been reported in neonates, but have been unusual in adults [2]. Catheters have been inserted into the inferior epigastric vein very rarely; only three cases have been reported in Japan (written in Japanese) as far as we know. Use of the inferior epigastric vein as an alternate route in children with difficult central venous access has been previously reported [3–5]. There are fewer complications associated with femoral vein catheter insertions than with either internal jugular vein or subclavian vein insertions. Infectious risk of catheter placement in femoral vein is higher compared to those in internal jugular vein or subcvlaian vein.

The indications for catheter placement for intravenous hyperalimentation are basically considered when the enteral nutrition is difficult for some reasons. The enteral nutrition should be considered as a first-option alternative for most of patients. In this case, the patient was planned to have the operation for a gastric fistula. Nasal feeding tube was not inserted, because the patient was easy to have epistaxis. 

Despite we initially confirmed catheter position by plain radiography, we could not demonstrate the displacement. Catheter migration was not confirmed by plain radiography in any of the previously reported cases. The cellulitis in our case completely resolved in a few days after catheter withdrawal. Two of the previously reported cases needed operations for wound drainage (cases written in Japanese).

In conclusion, the possibility in adults as well as children of intravenous catheters migrating into smaller vessels, such as the superficial and inferior epigastric veins, should be recognized after insertions into the femoral vein.
